# Changes in infant segment inertias during the first three months of independent walking

**DOI:** 10.1186/1476-5918-4-9

**Published:** 2005-10-28

**Authors:** Victoria L Chester, Robert K Jensen

**Affiliations:** 1 Faculty of Kinesiology, University of New Brunswick, Peter Kelly Drive, Fredericton, NB, Canada; 2 Human Movement Studies, Laurentian University, Sudbury, ON, Canada

## Abstract

**Background:**

During infancy, rapid changes in physical growth affect the size and shape of the body segments. To understand the effects of growth on movement, it is first necessary to quantify rates of development during the acquisition of important motor milestones. The goal of this longitudinal study was to quantify the physical growth of infant body segments during the initial stages of independent walking.

**Methods:**

Ten infants (N = 10) aged between 28 and 55 weeks at the beginning of the study were tested biweekly (every two weeks) for three months. A 13-segment mathematical model of the human body was used to estimate the inertial parameters of the infant body segments at each session. An analysis of variance was used to test for significant differences in segment masses between biweekly measures. Polynomial contrasts were used to test for linear trends in the growth data.

**Results:**

Significant differences between biweekly measures of segment mass were found only for the head/neck (F(5,45) = 3.42, p < 0.05), upper trunk (F(5,45) = 4.04, p < 0.01), and lower trunk (F(5,45) = 3.49, p < 0.01). The lower trunk demonstrated a linear increase in mass (F(1,9) = 4.56, p < 0.05). However, the upper trunk demonstrated a quadratic trend in growth (F(1,9) = 9.13, p < 0.01), while the head/neck segment showed a cubic trend in growth (F(1,9) = 3.80, p < 0.05). Significant differences in axial segment masses were also found between subjects (F(9,45) = 5.92, p < 0.001).

**Conclusion:**

Given that postural control proceeds in a cephalocaudal manner, the lower trunk segment would be brought under control last, in terms of the axial segments. Increases in the mass of this segment could constrain the system, thereby acting as a control parameter for the onset and development of motor patterns.

## Background

Current views of infant motor development consider movement to be the result of the collective and cooperative influence of both neural and non-neural elements, including physical growth, neuromaturation, arousal level, and environmental contexts. A principled account of motor development from this perspective can be found in dynamic systems theory [1-3]. In this theory, changes in segment inertial parameters are regarded as important system constraints or control parameters, involved in determining the emergence of new and progressively more stable infant motor patterns. This view is supported by evidence that shows segment inertial parameters have a substantial effect on infant motor patterns [4,5]. However, the examination of time-dependent changes in segment inertias is necessary to identify the mechanisms by which these physical elements facilitate shifts into new motor patterns.

During infancy, rapid changes in physical growth affect the size and shape of the body segments. These changes in segment mass and distribution of mass (moments of inertia) are partially consistent with the principles of cephalocaudal and distal to proximal development [6,7], as indicated by differential growth rates between infant body segments [8]. Differential growth rates of infant body segments could play a major role in the development of various motor skills. This longitudinal study aimed to quantify the changes in physical growth during the first three months of independent walking. During this time, infants experience rapid changes in growth, as well as improvements in the quality of movement. Examining the rates of growth enables the investigation of mechanisms by which segment inertial parameters may act as control parameters of movement.

## Methods

Ten healthy, full-term infants (6 males, 4 females) participated, aged between 28 and 55 weeks at the beginning of the study. To ensure the onset of independent walking occurred during the study period, infants capable of standing alone or with support, but unable to accomplish three independent steps, were selected. The infants were photographed, measured, and video-recorded biweekly (every two weeks). After the onset of independent walking, infants were required to maintain the biweekly visits for 3 months, for a total of 6 repeated measures. At the end of the study the age range of the infants was 48 to 68 weeks. The Laurentian University Centre for Research in Human Development approved the procedures employed and written informed consent was obtained from the parents' of the participants.

**Figure 1:**
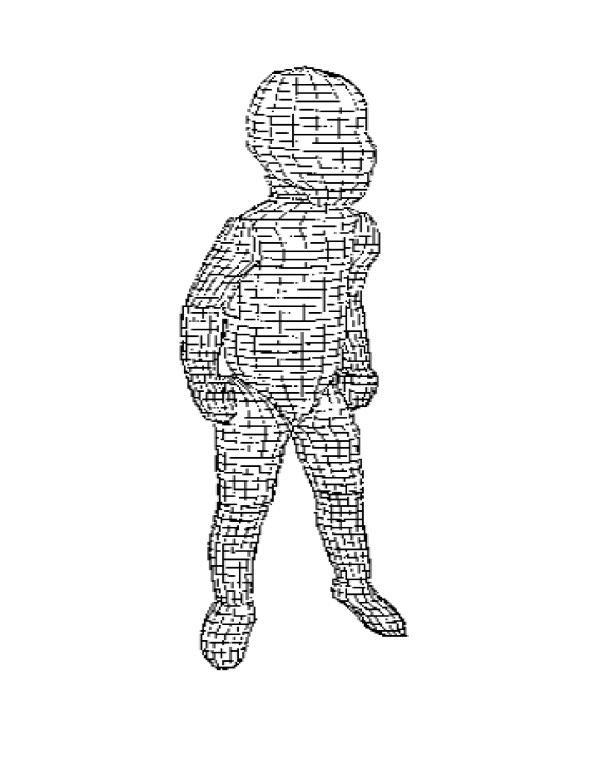
Elliptical cylinder model of an infant.

Infant segment inertial parameters were estimated using a mathematical model of the body [8] with minor modifications. The model consisted of 13 segments, specifically the head/neck (combined), upper trunk, lower trunk, arm, forearm/hand (combined), thigh, shank, and foot. Each segment was assumed to consist of stacked right elliptical cylinders sectioned at regular 1.0-cm intervals in the transverse plane (Figure 1). As infant segment densities are unavailable, adult segment densities were used [9]. Each infant was suspended vertically in a harness and photographed using two 35-mm cameras placed orthogonally to obtain front and side views. The images of the body and joint centres were projected, outlined, and then digitised. Segment lengths, radii to the centres of mass, masses, and principal moments of inertia were calculated from the stacked elliptical cylinders and joint centres representing each segment [10,11].

Infant body segments showing significant differences in mass between biweekly sessions were identified using a one-way ANOVA. Polynomial contrasts were used to test for linear trends in the growth of these segments over the three month period.

## Results

The elliptical cylinder model yielded a small underestimation of total body mass. The mean error between the actual and estimated total body mass was -0.04%, with a standard deviation of 2.73%. The means and standard deviations of the axial segment masses from the 6 repeated measures are provided in Table 1.

**Table 1:** Means and standard deviations of segment masses (kg) over the three month study.

Segment	Week 1	Week 3	Week 5	Week 7	Week 9	Week 11
Head/Neck	2.40 (0.25)	2.32 (0.17)	2.43 (0.23)	2.28 (0.30)	2.16 (0.25)	2.36 (0.31)
Upper Trunk	1.68 (0.24)	1.46 (0.23)	1.60 (0.20)	1.70 (0.25)	1.58 (0.16)	1.48 (0.12)
Lower Trunk	2.09 (0.25)	2.48 (0.48)	2.49 (0.62)	2.46 (0.32)	2.63 (0.40)	2.57 (0.49)

**Figure 2:**
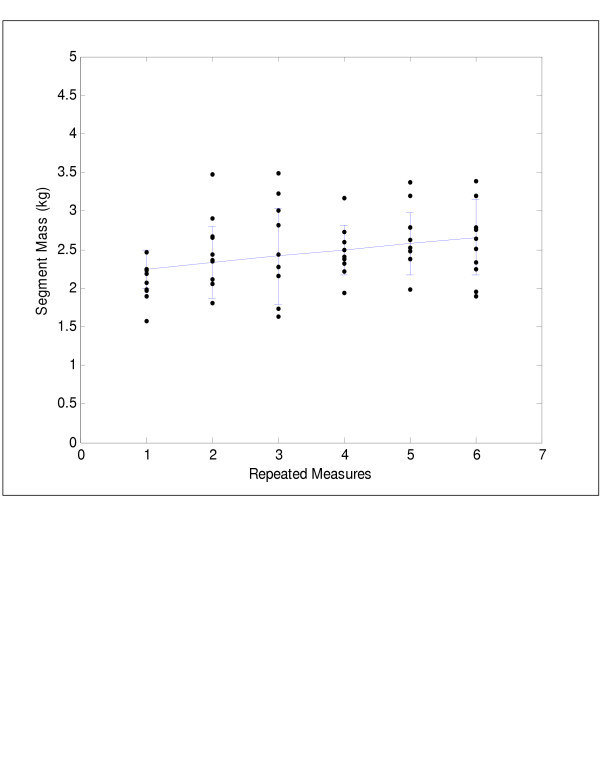
Lower trunk segment mass versus time showing the linear trend in growth.

**Figure 3:**
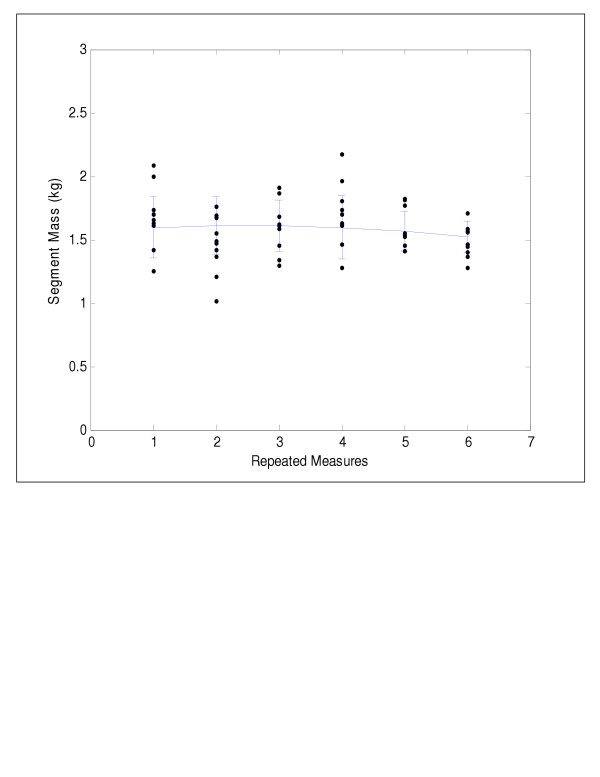
Upper trunk segment mass versus time showing a quadratic trend in growth.

**Figure 4:**
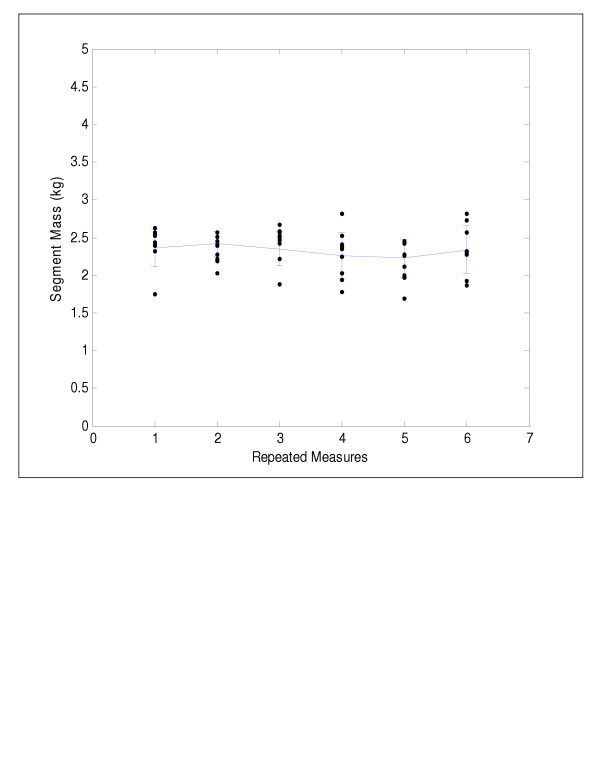
Head/neck segment mass versus time showing a cubic trend in growth.

Significant differences between biweekly measures of segment mass were found only for the head/neck (F(5,45) = 3.42, p < 0.01), upper trunk (F(5,45) = 4.04, p < 0.01), and lower trunk (F(5,45) = 3.49, p < 0.01). The mean growth velocities of these axial segments are depicted in Figures 2, 3, 4. The lower trunk demonstrated an increase in mass, while the upper trunk and head/neck segments demonstrated non-linear changes in mass over the three month period. Polynomial contrasts revealed a linear growth pattern for the lower trunk segment only (F(1,9) = 4.56, p < 0.05). The upper trunk demonstrated a quadratic trend in growth (F(1,9) = 9.13, p < 0.01), while the head/neck segment showed a cubic trend in growth (F(1,9) = 3.80, p < 0.05). Significant differences in segment growth between children (F(9,45) = 5.92, p < 0.001) were also observed for the axial segments and were likely due to differences in age and rates of development.

## Discussion

Changes in segment inertial parameters are regarded as control parameters, involved in determining the emergence of new and progressively more stable infant motor patterns. The examination of time-dependent changes in segment inertias may increase our understanding of the relationships between physical growth and infant motor development. The present study identified significant changes in mass of the axial segments during the first three months of independent walking.

The accuracy of the elliptical cylinder model was evaluated using the total body mass estimation. The mean error of the estimated infant segment inertial parameters was small and comparable to previous studies (Sun & Jensen, 1994: M = 2.27%, S.D. = 3.85%; Schneider & Zernicke, 1992: M = -0.14%, S.D. = 4.27%).

Significant differences between biweekly measures of mass were found only for the axial segments. The lower trunk demonstrated a significant linear increase in mass, while the upper trunk and head/neck segments showed significant non-linear changes in mass over the three month period. These latter two segments showed periods of increasing and decreasing mass. The pattern of change in these segment masses was consistent with the principle of cephalocaudal development [7], whereby the rate of growth would be initially high in the head segment and peak progressively distally. Considering postural control also proceeds in a cephalocaudal manner, it is possible that the inertial parameters of the lower trunk segment specifically, are affecting the acquisition and stability of independent walking patterns. This segment, which would be brought under control last, may constrain the system and act as a control parameter. Small changes in the inertial parameters of the lower trunk with development could lead to a new balance between gravitational and muscular joint moments, thereby facilitating the emergence and stabilization of walking patterns.

## Conclusion

We contend that the growth velocity of the lower trunk should be regarded as a potential control parameter of activities such as walking. Further research efforts should be directed towards perturbing these elements to engender behaviour changes in real-time and to verify their roles as control parameters within a movement context.

## Authors' contributions

VLC carried out the data collection and data analysis. RKJ participated in the design of the study. RKJ and VLC conceived of the study, and RKJ participated in its design and coordination and helped to draft the manuscript. All authors read and approved the final manuscript.
